# Mice with missense and nonsense *NF1* mutations display divergent phenotypes compared with human neurofibromatosis type I

**DOI:** 10.1242/dmm.025783

**Published:** 2016-07-01

**Authors:** Kairong Li, Ashley N. Turner, Min Chen, Stephanie N. Brosius, Trenton R. Schoeb, Ludwine M. Messiaen, David M. Bedwell, Kurt R. Zinn, Corina Anastasaki, David H. Gutmann, Bruce R. Korf, Robert A. Kesterson

**Affiliations:** 1 Department of Genetics, The University of Alabama at Birmingham, Birmingham, AL 35294, USA; 2 Medical Scientist Training Program, The University of Alabama at Birmingham, Birmingham, AL 35294, USA; 3 Department of Biochemistry and Molecular Genetics, The University of Alabama at Birmingham, Birmingham, AL 35294, USA; 4 Department of Radiology, The University of Alabama at Birmingham, Birmingham, AL 35294, USA; 5 Department of Neurology, Washington University School of Medicine, St. Louis, MO 63110, USA

**Keywords:** Neurofibromatosis type 1, Patient-derived mouse models, Nonsense mutation, Missense mutation

## Abstract

Neurofibromatosis type 1 (NF1) is a common genetic disorder characterized by the occurrence of nerve sheath tumors and considerable clinical heterogeneity. Some translational studies have been limited by the lack of animal models available for assessing patient-specific mutations. In order to test therapeutic approaches that might restore function to the mutated gene or gene product, we developed mice harboring NF1 patient-specific mutations including a nonsense mutation (c.2041C>T; p.Arg681*) and a missense mutation (c.2542G>C; p.Gly848Arg). The latter is associated with the development of multiple plexiform neurofibromas along spinal nerve roots. We demonstrate that the human nonsense NF1^Arg681*^ and missense *NF1^Gly848Arg^* mutations have different effects on neurofibromin expression in the mouse and each recapitulates unique aspects of the NF1 phenotype, depending upon the genetic context when assessed in the homozygous state or when paired with a conditional knockout allele. Whereas the missense *Nf1^Gly848Arg^* mutation fails to produce an overt phenotype in the mouse, animals homozygous for the nonsense Nf1^Arg681*^ mutation are not viable. Mice with one Nf1^Arg681*^ allele in combination with a conditional floxed *Nf1* allele and the *DhhCre* transgene (Nf1^4F/Arg681*^; DhhCre) display disorganized nonmyelinating axons and neurofibromas along the spinal column, which leads to compression of the spinal cord and paralysis. This model will be valuable for preclinical testing of novel nonsense suppression therapies using drugs to target in-frame point mutations that create premature termination codons in individuals with NF1.

## Abstract (precis)

**Summary:** Two mouse models carrying NF1 patient-specific mutations have distinct effects on embryonic development, neurofibromin function and plexiform neurofibroma formation, with implications for therapeutic studies.

## INTRODUCTION

Neurofibromatosis type 1 (NF1) is a common genetic disorder affecting ∼1 in 3000 individuals and over two million people worldwide (Friedman, 1999; Evans et al., 2010). NF1 is characterized principally by the occurrence of neurofibromas and café-au-lait macules (CALMs) (Boyd et al., 2009). Both children and adults with NF1 are at an increased risk for developing certain malignancies and display an assortment of benign and malignant lesions including neurofibromas (dermal and plexiform), pheochromocytomas, pilocytic astrocytomas, optic gliomas, bone abnormalities, leukemias and malignant peripheral nerve sheath tumors (Boyd et al., 2009). NF1 is caused by inherited or *de novo* pathogenic variants in the *NF1* gene, whereas tumor development requires additional mutation of the other *NF1* allele (loss of heterozygosity) (Garcia-Linares et al., 2011; Steinmann et al., 2009). To date, no strong genotype-phenotype correlation has been established for NF1 because of the varied disease expression observed in humans. Many individuals with NF1, even from the same family, show differences in the presentation or severity of the NF1 phenotype (Pasmant et al., 2012; Alkindy et al., 2012; Sabbagh et al., 2009). This is exemplified by the National Institutes of Health (NIH) diagnostic criteria for NF1, which require a minimum of two of the following: six or more CALMs, two or more cutaneous neurofibromas or one plexiform neurofibroma, axillary or inguinal freckling, optic pathway glioma, two or more Lisch nodules, bony dysplasia, or a first degree relative with NF1 (Ferner et al., 2007). Furthermore, there are few *in vitro* assays available to examine neurofibromin function, which is especially problematic when trying to assess the potential pathogenicity of missense variants.

Several mouse models have been developed that display some of the phenotypes seen in individuals with NF1, including learning difficulties, as well as the development of plexiform neurofibromas, optic gliomas and malignant peripheral nerve sheath tumors (Boyd et al., 2009). These existing animal models generally employ conditional knockout strategies that lead to loss of function of one or both *Nf1* alleles, which have proven to be valuable tools to investigate the pathogenesis of NF1, and to evaluate several therapeutic strategies such as inhibitors of RAS or intercellular signaling (Yang et al., 2008; Robertson et al., 2012; Jessen et al., 2013). However, the underlying mutations used in current animal models are not sufficient to evaluate novel therapeutic approaches to target the mutated gene or gene product, including nonsense suppression and exon-skipping therapies. These therapies have been established in Duchenne muscular dystrophy and cystic fibrosis (Du et al., 2002, 2006; De Luca et al., 2008; Loufrani et al., 2004; Barton-Davis et al., 1999; Zhang et al., 2014; Xue et al., 2014; Mann et al., 2001; Lu et al., 2005; van Deutekom et al., 2001; Aartsma-Rus et al., 2003; Igreja et al., 2016). Thus, the use of NF1 mouse models harboring mutations identified in individuals with NF1 provides the ability to perform prospective investigations targeting a specific underlying mutation or mutation type.

To this end, we created and characterized two novel mouse models harboring mutations found in individuals with NF1, including a common nonsense mutation (nmNF1, exon 18 c.2041C>T; p.Arg681*) expected to produce complete loss of function, and a recurring missense mutation found in individuals with NF1 associated with multiple plexiform neurofibromas along spinal nerve roots (exon 21 c.2542G>C; p.Gly848Arg). We demonstrate that the nonsense c.2041C>T; p.Arg681* (hereafter called Nf1^Arg681*^) and missense c.2542G>C; p.Gly848Arg (hereafter called *Nf1^Gly848Arg^*) mutations in the mouse have different effects on neurofibromin expression, and each recapitulates unique aspects of NF1 depending upon the genetic context when assessed in the homozygous state, or when paired with a conditional knockout allele.

## RESULTS

### Establishing mouse models with NF1 patient mutations

Nonsense mutations make up ∼20% of characterized mutations (Messiaen and Wimmer, 2008). The Medical Genomics Laboratory at the University of Alabama at Birmingham has identified 19 of the most recurrent nonsense mutations found in individuals with NF1 (each responsible for at least 0.3% of NF1 cases). Among those is a nonsense mutation in exon 18, c.2041C>T; p.Arg681* observed in 49/8000 unrelated *NF1*-mutation-positive probands (0.61%). Of 39 individuals with phenotypic data available, 15 individuals were 0-2 years of age at the time of testing (mostly too young to fulfill clinical diagnostic criteria). Within these individuals, 2/15 fulfill NIH criteria with 2/15 individuals having <6 CALMs. The remaining 24 individuals are ≥3 years of age and all fulfill NIH criteria for NF1 diagnosis; although none possess symptomatic spinal neurofibromas (including half who were ≥19 years old), 8/24 individuals did have an externally visible plexiform neurofibroma.

In contrast, a missense mutation located in exon 21 (c.2542G>C; p.Gly848Arg), observed in 11/7800 unrelated *NF1*-mutation-positive probands (0.14%), is associated with symptomatic spinal nerve root tumors. Phenotypic data were obtained from 20 individuals from four different families with a p.Gly848Arg mutation. Three of these individuals are 0-2 years of age, with 1/3 fulfilling the NIH criteria and 2/3 having <6 CALMs, whereas 7/17 of the individuals aged ≥3 years fulfill the NIH criteria, with 4/17 having an externally visible plexiform neurofibroma. Symptomatic spinal nerve root tumors were found in 6/17 individuals, including 5/8 individuals who were ≥19 years old. For individuals 19 years or older, there is a significant difference in the frequency of spinal nerve root tumors between the c.2041C>T (0/12) vs c.2542G>A (5/8) mutation (2-tailed Fisher exact: *P*=0.0036).

To model each human mutation in the mouse, knock-in animals harboring the nonsense c.2041C>T; p.Arg681* mutation allele in exon 18 (Nf1^Arg681*^) and the missense c.2542G>C; p.Gly848Arg mutation allele in exon 21 (*Nf1^Gly848Arg^*) were created using conventional gene-targeting vectors and C57BL/6 embryonic stem cells, with chimeric mice and germ line transmission being confirmed (Table S1). Since the *Nf1*-floxed animals that have been used in previous studies were generated on a mixed genetic background (Zhu et al., 2002), we obtained mice from the International Knockout Mouse Consortium (IKMC) harboring a ‘knockout first’ allele for the *Nf1* gene (*Nf1^tm1a(KOMP)Wtsi^*) in order to have all *Nf1* alleles on the inbred C57BL/6 background. We generated a new conditional knockout ‘floxed’ allele of the *Nf1* gene where exon 4 is flanked by loxP sites (hereafter referred to as *Nf1^4F^*) after removal of the FRT-flanked LacZ/Neo cassettes by breeding to a FLP-recombinase line. Matings between heterozygous *Nf1^+/4F^* mice yielded homozygous floxed *Nf1^4F/4F^* mice at the expected Mendelian ratio that were viable, fertile and did not show gross or histological abnormalities (data not shown). Heterozygous *Nf1*^+/^*^Δ4^* mice harboring a null allele resulting from exon 4 deletion were created by breeding *Nf1^+/4F^* mice to an EIIaCre driver line [B6.FVB-Tg(EIIa-cre)C5379Lmgd/J strain #003724], thereby deleting the floxed sequence in all cells within the developing embryos.

### Nonsense mutation NF1^Arg681*^ recapitulated in the mouse is non-functional

**Fig. 1.**
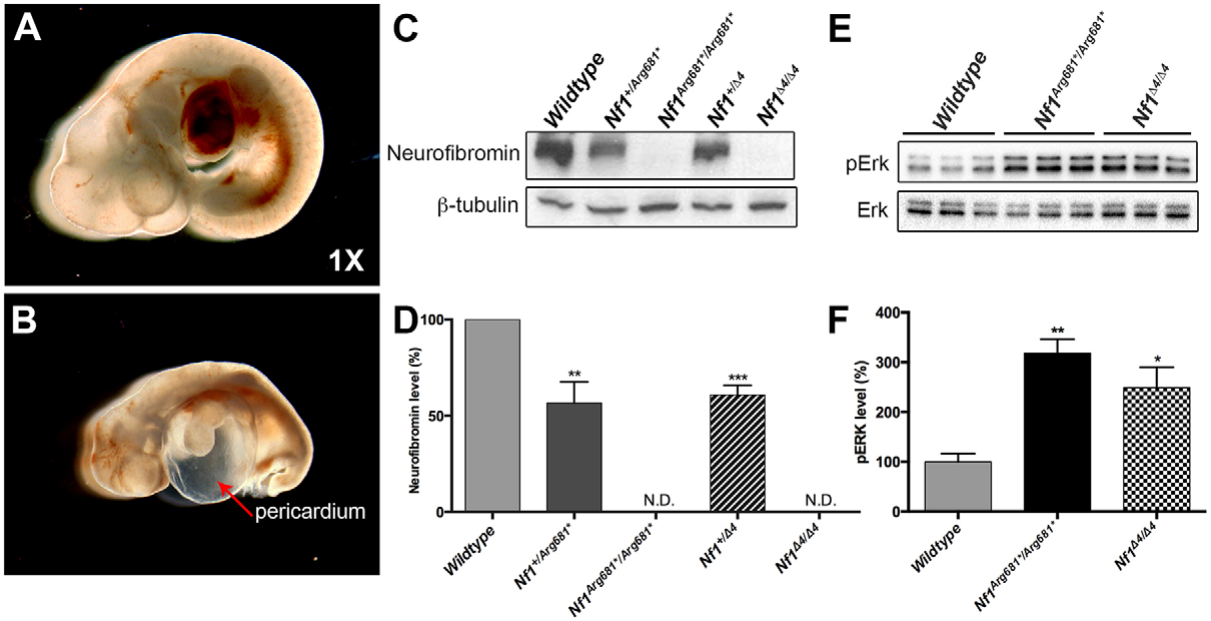
Nf1^Arg681*/Arg681*^ mice fail to develop after E9.5. (A,B) Morphology of representative wild-type (A) and Nf1^Arg681*/Arg681*^ (B) embryos at E10.5. The red arrowhead in B indicates effusion into the pericardial space. The magnification is noted in the lower right corner. (C-F) Western blot of neurofibromin (C) and phosphorylated ERK (pERK) (E) in E9.5 MEFs of indicated genotypes. Results are representative of three independent experiments or samples. β-tubulin is shown as loading control. The western blot analysis of neurofibromin (D) and pERK (F) was quantified, and the results are expressed in histograms. *P<0.05, **P<0.01, ***P<0.001 vs wild type by one-way ANOVA. Data are presented as mean±s.e.m. MEFs, mouse embryonic fibroblasts. N.D., none detected.

Previous studies have demonstrated that mouse embryos fail to develop beyond embryonic day (E)13.5 in the absence of *Nf1* expression as a result of cardiovascular defects (Jacks et al., 1994; Brannan et al., 1994). To determine the functionality of each allele, we performed intercross breedings of Nf1^+/Arg681*^ and *Nf1*^+/^*^Δ4^* mice and examined embryos from E9.5 to E13.5. Embryos heterozygous for either the Nf1^Arg681*^ or the *Nf1^Δ4^* allele were viable and developed to term with no observable defects. At E9.5, all homozygous Nf1^Arg681*/Arg681*^ and *Nf1^Δ4^*^/^*^Δ4^* embryos were indistinguishable from their littermate controls; however, these homozygous embryos fail to develop past this point (Fig. S2). Although Nf1^Arg681*/Arg681*^ mice display pericardial effusions at E10.5 (Fig. 1A,B), we were unable to ascertain the exact cause of death because of extensive tissue degradation.

To assess neurofibromin function in these models, we isolated and expanded mouse embryonic fibroblasts (MEFs) from E9.5 embryos carrying different allelic mutations, and performed western blot analyses. Neurofibromin was completely absent in Nf1^Arg681*/Arg681*^ and *Nf1^Δ4^*^/^*^Δ4^* MEFs, and was markedly reduced in Nf1^+/Arg681*^ and *Nf1*^+/^*^Δ4^* MEFs (Fig. 1C,D). Neurofibromin negatively regulates Ras signaling by accelerating conversion of activated Ras-GTP to inactive Ras-GDP, resulting in hyperactivation of phosphorylated ERK (pERK). We therefore compared pERK levels in wild-type, Nf1^Arg681*/Arg681*^ and *Nf1^Δ4/Δ4^* MEFs derived from E9.5 embryos, and observed significant elevations in pERK levels in Nf1^Arg681*/Arg681*^ and *Nf1^Δ4^*^/^*^Δ4^* MEFs as compared with wild-type controls (Fig. 1E,F). Both Nf1^Arg681*/Arg681*^ and *Nf1^Δ4^*^/^*^Δ4^* genotypes produced comparable alterations in neurofibromin and pERK expression. Taken together, these data provide evidence that Nf1^Arg681*^ functions as a null allele.

Nonsense mutations create premature termination codons (PTCs) in genes that in turn trigger nonsense-mediated mRNA decay (NMD) to selectively and rapidly degrade PTC-bearing aberrant transcripts. To determine if Nf1^Arg681*^ mutations induce NMD, we compared levels of wild-type versus mutant mRNA in Nf1^+/Arg681*^ mice. It is expected that in the absence of NMD, the percentage ratio of mutant to wild-type mRNA would be 50:50; however, if NMD were occurring, the distribution of mRNA would be skewed. RNA was extracted from brain tissues of Nf1^+/Arg681*^ mice and reverse transcription polymerase chain reaction (RT-PCR) was performed using primer sets flanking the nonsense mutation. The amplicons were then cloned into a TA vector for sequencing. Of twenty-two clones sequenced, only two (9.1%) were identified with the Nf1^Arg681*^ mutation whereas the remaining twenty clones (90.9%) contained wild-type sequences. This skewed and unequal representation of wild-type versus mutant clones (2-tailed Fisher exact; *P*=0.0068) indicates the occurrence of NMD of the Nf1^Arg681*^ allele transcripts. These findings indicate that both Nf1^Arg681*^ and the novel *Nf1^Δ4^* alleles significantly ablate *Nf1* gene expression and function.

### In the appropriate genetic context, the Nf1^Arg681*^ mutation induces neurofibroma formation in mice

As described above, individuals with NF1 harboring nonsense NF1^Arg681*^ mutations may develop plexiform neurofibromas; therefore, we hypothesized that we could recapitulate this phenotype in Nf1^Arg681*^ mice in the appropriate genetic context. Using conditional knockout models, it has been established that neurofibroma development can be stimulated through *Nf1* loss of heterozygosity within the Schwann cell lineage when driven by Cre recombinase expression (Wu et al., 2008). To investigate whether the Nf1^Arg681*^ and the *Nf1^4F^* allele can induce neurofibroma development *in vivo*, we generated two conditional knockout NF1 mouse models employing a *DhhCre* transgenic mouse line. In this model system, Cre recombinase is expressed from *desert hedgehog* regulatory sequences at E12.5, with previous demonstration that inactivation of the *Nf1* gene in *Nf1^31&32F/31&32F^; DhhCre* mice (with exons 31 and 32 flanked by loxP sites) elicits the formation of plexiform and subcutaneous neurofibromas (Wu et al., 2008).

**Fig. 2.**
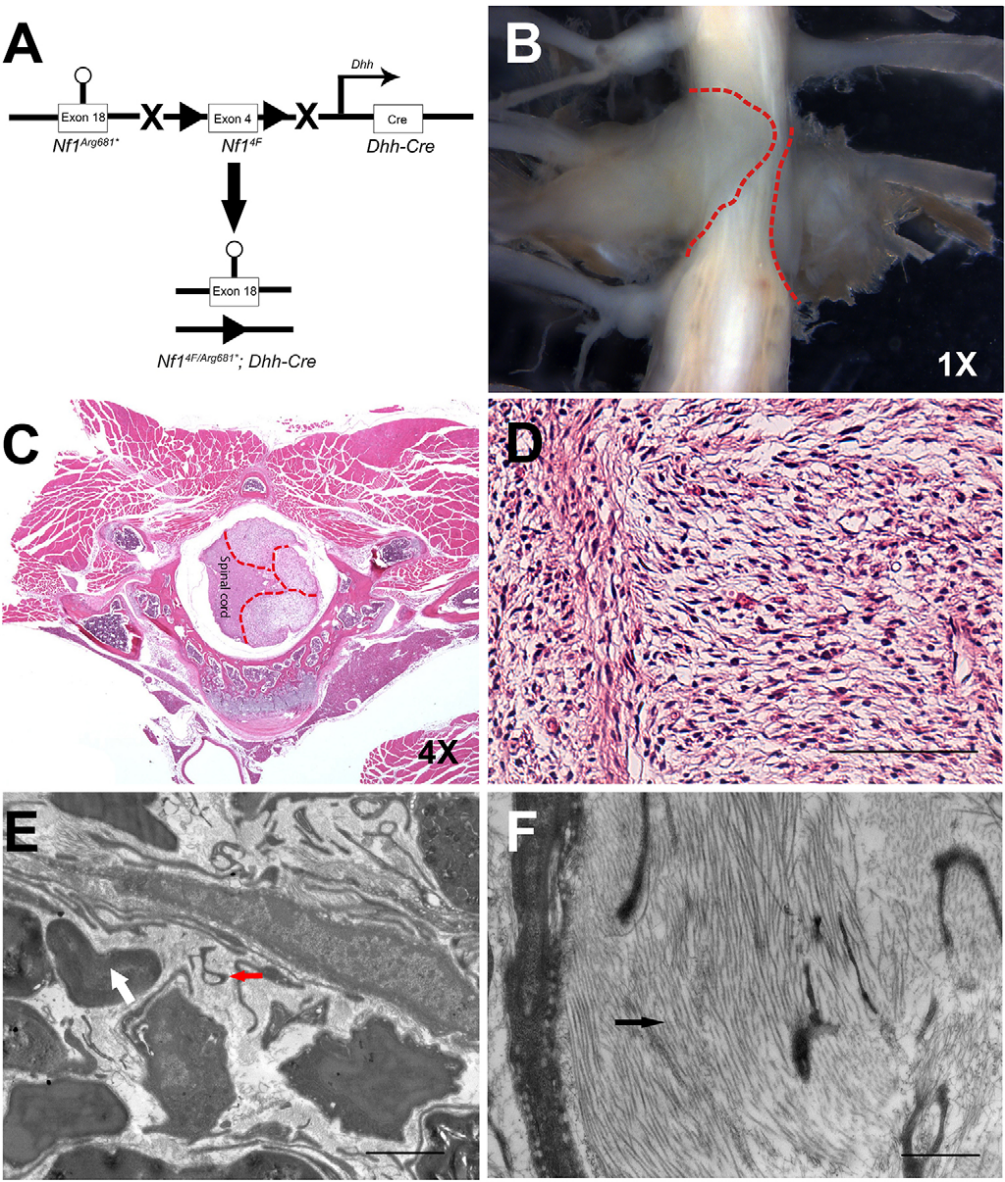
Nf1^4F/Arg681*^; Dhh-cre mice develop neurofibromas along the spinal column and display disrupted ultrastructure. (A) Schematic illustration of the strategy used to breed the Nf1^4F/Arg681*^; Dhh-Cre mice. The circle indicates the targeted mutation, triangles represent LoxP sites. (B-D) Gross dissection of spinal neurofibromas (B) and H&E staining (C) of neurofibromas along spinal cord of Nf1^Arg681*/Arg681*^; Dhh-Cre mice. Red dashed lines demarcate the neurofibroma boundaries. (D) A higher magnification demonstrating classic neurofibroma histology. (E,F) The aberrant ultrastructure of the neurofibromas as shown by electron microscopy of the Cre-negative control in (E) and the mutant in (F). The white arrow and red arrowhead indicate normal myelinated axons or disrupted nonmyelinating axons, respectively. The black arrowhead indicates collagen deposits. The magnifications are as indicated. Scale bars: 40 μm in D, 1 μm in E,F.

We bred the Nf1^+/Arg681*^, *Nf1^4F^*^/4F^ and *DhhCre* mice and generated Nf1^4F/Arg681*^; DhhCre mice (Fig. 2A) as well as *Nf1^4F/4F^; DhhCre* mice. Consistent with previous studies, both Nf1^4F/Arg681*^; DhhCre and *Nf1^4F/4F^; DhhCre* mice were viable and indistinguishable from their littermate controls at birth. However, we began to observe paralysis in one or both hind limbs in animals from both models at approximately four months of age. By five months of age, 55.6% (30/54) of our initial cohort of Nf1^4F/Arg681*^; DhhCre mice exhibited paralysis, whereas 54.1% (13/24) of *Nf1^4F/4F^; DhhCre* mice were paralyzed at the same age. In addition to paralysis, these mice also developed other symptoms including lethargy, weight loss, dermatitis and/or dehydration.

**Fig. 3.**
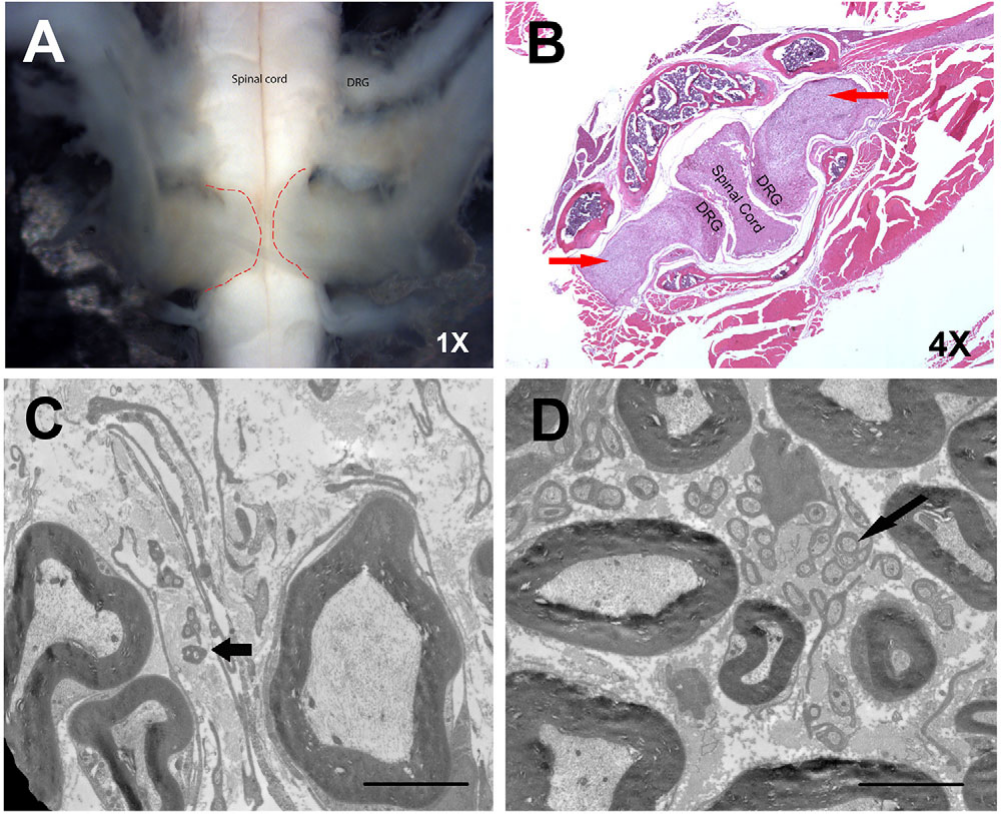
***Nf1^4F/4F^; DhhCre* mice develop neurofibromas and sciatic nerve lesions.** (A,B) Gross dissection (A) and H&E staining (B) of spinal tumors in *Nf1^4F/4F^; DhhCre* mice. Red dashed lines demarcate the neurofibroma boundaries. Red arrows indicate neurofibromas. (C,D) The ultrastructure, as shown by electron microscopy, shows similar lesions in the Nf1^4F/Arg681*^; DhhCre mice in both neurofibromas (C) and sciatic nerves (D). Black arrows indicate abnormal nonmyelinating axons. The magnifications are as indicated. Scale bars: 4 μm in C,D.

To determine the source of the paralysis, we performed gross dissection of each Nf1^4F/Arg681*^; DhhCre or *Nf1^4F/4F^; DhhCre* mouse. Tumors were observed in 81.5% (44/54) of Nf1^4F/Arg681*^; DhhCre and 79.2% (19/24) of *Nf1^4F/4F^; DhhCre* mice. All paralyzed mice were found to have visible tumors arising at the dorsal root ganglia resulting in compression of the spinal cord. Gross tumor numbers ranged from one to thirteen per mouse, with 90.9% detected in the cervical or thoracic spine and 9.1% detected in the lumbar spine (Fig. 2B, Fig. 3A). These tumors seemed to arise around the dorsal root ganglia, with invasion of the neural foramen, leading to compression of the spinal cord. In addition to these lesions, we also observed enlarged spinal nerve roots and peripheral nerves in the mice examined (data not shown). Thus, Nf1^4F/Arg681*^; DhhCre mice develop neurofibromas with high efficiency.

Upon histological analysis, neurofibromas are characterized by disorganized Remak bundles (the non-myelinated axon-Schwann cell unit) and enriched deposition of collagen. Similar lesions were seen from tumor preparations of both models at the ultrastructural level (Fig. 2C-F, Fig. 3B-D). These data indicate that Nf1^Arg681*^ functions as a null allele, and recapitulates key characteristics of human disease in the proper genetic context.

### Missense mutation *NF1^Gly848Arg^* recapitulated in the mouse retains function

**Fig. 4.**
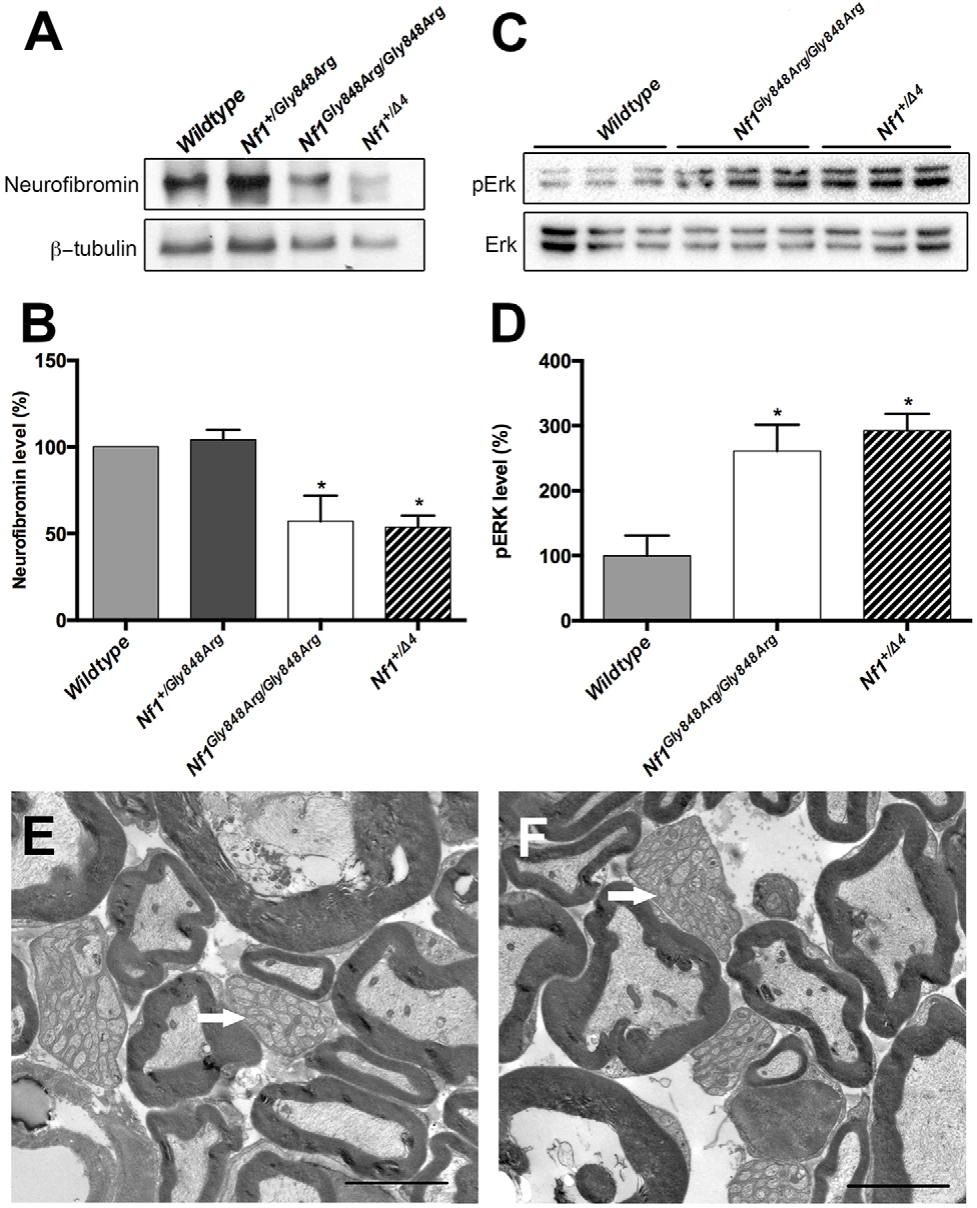
**The missense *Nf1^Gly848Arg^* allele impairs neurofibromin expression and function.** (A-D) Western blots of neurofibromin (A) and pERK (C) in E13.5 MEFs from three independent experiments. β-tubulin is shown as loading control. The western blot analysis of neurofibromin (B) and pERK (D) was quantified and the results expressed in histograms. (E,F) Electron micrographs from spinal nerve roots of control (E) and *Nf1^Gly848Arg/Gly848Arg^* (F) mice. White arrows represent normal nonmyelinating axons. *P<0.05 vs wild type by one-way ANOVA. Data are presented as mean±s.e.m. Scale bars: 4 μm in E,F.

Mice homozygous for the *Nf1^Gly848Arg^* missense allele (*Nf1^Gly848Arg/Gly848Arg^*) are viable and are indistinguishable from their heterozygous (*Nf1^+/Gly848Arg^*) siblings. A cohort of 19 *Nf1^Gly848Arg/Gly848Arg^* mice were monitored throughout their lifetimes for tumor development. No overt phenotype was observed. All animals underwent gross dissection, and we were unable to detect tumors in the spinal columns or the peripheral nerves. The distinct manifestations between *Nf1^Gly848Arg^* and Nf1^Arg681*^ might suggest different underlying *Nf1* gene expression and function. We therefore derived MEFs from E13.5 embryos and assessed neurofibromin expression by western blot (Fig. 4A). The *Nf1^Gly848Arg^* allele impairs neurofibromin expression, as *Nf1^Gly848Arg/Gly848Arg^* MEFs show ∼50% less protein than cells from wild-type controls, which is similar to that observed in *Nf1^+/Δ4^* samples (Fig. 4B). In response to neurofibromin reduction, pERK is hyperactivated in *Nf1^Gly848Arg/Gly848Arg^* MEFs to a level comparable with *Nf1^+/Δ4^* samples, suggestive of increased Ras activity (Fig. 4C,D). Therefore, *Nf1^Gly848Arg^* only partially decreases *Nf1* gene expression in mouse. Given that spinal neurofibromas form the primary manifestation of this mutation in humans, we examined the spinal nerve roots of *Nf1^Gly848Arg/Gly848Arg^* mice by electron microscopy for ultrastructural abnormalities. Neither myelinated nor non-myelinated axons were disrupted when compared with controls. (Fig. 4E,F).

Individuals with NF1 carry second (somatic) mutations that are distinct from germline mutations. We hypothesized that *Nf1^Gly848Arg^* mice carrying a second null mutation in *Nf1* might develop neurofibromas, which we tested by generating *Nf1^Δ4^*^/*Gly848Arg*^ mice. Similar to *Nf1^Gly848Arg/Gly848Arg^* mice, these animals were viable and displayed no overt phenotype. We monitored ten *Nf1^Δ4^*^/*Gly848Arg*^ mice for up to twenty-four months and still were unable to find tumors. Therefore, we conclude that the missense mutation *Nf1^Gly848Arg^* only partially impacts *Nf1* gene function, and is unable to induce neurofibroma development in mice.

## DISCUSSION

The factors that underlie the clinical heterogeneity seen in NF1 are still unclear. Assessment of the functional and physiological consequences of putative mutations is hampered by the lack of robust *in vitro* and *in vivo* assays, and limited knowledge of neurofibromin protein domains. In this study, two animal models were engineered to harbor *NF1* mutations found in human patients, specifically nmNF1 (nonsense c.2041C>T; p.Arg681*) and a missense mutation of NF1 (missense c.2542G>C; p.Gly848Arg). This approach is possible given the greater than 98% amino acid sequence homology between the mouse and human neurofibromin protein (Bernards et al., 1993). Within the regions encoded by exons 18 and 21, the protein sequence homology is 98% and 96%, respectively (Fig. S3). The clinical features associated with these mutations in humans are distinct. Individuals with a p.Gly848Arg mutation suffer from multiple plexiform neurofibromas along the spinal cord but a paucity of dermal neurofibromas, whereas individuals with the c.2041C>T; p.Arg681* premature stop mutation also have dermal and plexiform neurofibromas but do not have multiple spinal nerve root tumors.

Examining the effects of these two different *NF1* mutations in mouse models, we would like to make four points. Firstly, the two mutations have distinct effects on embryonic development and neurofibromin expression in the mouse. The Nf1^Arg681*^ allele functions as a null allele with complete loss of *Nf1* function, whereas *Nf1^Gly848Arg^* appears to retain some function. The Nf1^Arg681*^ allele is embryonic lethal when homozygous, whereas *Nf1^Gly848Arg^* homozygous animals are viable. In accordance with embryonic lethality, complete ablation of neurofibromin expression was observed in Nf1^Arg681*/Arg681*^ cells whereas neurofibromin expression in the *Nf1^Gly848Arg/Gly848Arg^* MEFs was reduced by ∼50%. Our data are consistent with a previous study on iPS cells and fibroblasts from individuals with NF1, which demonstrated that different germline *NF1* mutations result in different degrees of neurofibromin expression (Anastasaki et al., 2015). However, *in vitro* analyses showed increases in pERK in MEFs from both homozygotes when compared with wild-type. This is comparable with what has been observed in neural progenitor cells from individuals with NF1, as individual *NF1* mutations did not differentially affect Ras activation (Anastasaki et al., 2015). The observed changes in pERK suggest that the *Nf1^Gly848Arg^* allele could potentially function as a hypomorphic allele, though further studies would be necessary to confirm this hypothesis.

Secondly, the *Nf1^Gly848Arg^* allele failed to recapitulate the NF1 phenotype; whereas humans with NF1 suffer from multiple spinal plexiform neurofibromas, the mouse did not display an overt tumorigenic phenotype. A possible explanation for this discrepancy is the presence of modifier genes in the mouse impeding neurofibroma formation. In contrast, the Nf1^Arg681*^ allele stimulates tumorigenesis when placed in context with a loss-of-function allele as demonstrated with the Nf1^4F/Arg681*^; DhhCre mice. Similar observations were made in a parallel study where the same Nf1^Arg681*^ allele in a different mouse model led to development of optic gliomas, but these tumors were not seen in *Nf1^Gly848Arg^* mice (Toonen et al., 2016).

Thirdly, the Nf1^4F/Arg681*^; DhhCre model displays a robust tumor phenotype, with 81.5% of animals grossly developing neurofibromas throughout their lifetime. Previous studies using the *Nf1^31&32F/31&32F^*; *DhhCre* model indicated that mice became paralyzed in one or both hind limbs in 28.6% of animals by six months of age (Wu et al., 2008); however, our data with the Nf1^4F/Arg681*^; DhhCre model reveals an earlier onset of paralysis with tumorigenesis at 5 months of age in 55.6% of mice. These differences may either stem from the Nf1^Arg681*^ allele, which provides a suitable environment for neurofibroma development, or from the conditional knockout of exon four versus the previously described exon 31 and 32, as the *Nf1^4F/4F^; DhhCre* mice also appear to exhibit higher penetrance than the existing *Nf1^31&32F/31&32F^*; *DhhCre* model. This hypothesis is further supported by the notable differences in embryonic lethality between the *Nf1^Δ4^* allele, Nf1^Arg681*^ and the previously published *Nf1^31&32^*^F^ allele. As previously reported, embryonic death of *Nf1^Δ31&32/Δ^*^31&32^ animals occurred from E11.5 to E13.5 as a result of defects in cardiac development (Jacks et al., 1994; Brannan et al., 1994). However, all embryos carrying the newly developed *Nf1^Δ4/Δ4^* or Nf1^Arg681*/Arg681*^ died around E10. Additionally, a parallel study observed that optic gliomas from mice harboring the Nf1^Arg681*^ allele are more aggressive than those developing in mice with the traditional *Nf1^Δ31&32/Δ^*^31&32^ mutation, with more microglia and increased proliferative indices (Toonen et al., 2016). While it is difficult to make direct comparisons and draw conclusions across these studies because of the difference in null alleles utilized, collectively these data suggest the *Nf1^Δ4^* and Nf1^Arg681*^ alleles might yield a more severe phenotype.

Another possibility for the observed differences between *Nf1^4F/4F^; DhhCre* and *Nf1^31&32F/31&32F^*; *DhhCre* mice lies in the strains utilized in these studies; all mice in our experiments are on an inbred C57BL/6 background whereas previous studies used mice on a mixed 129/C57BL/6 background (Wu et al., 2008). Furthermore, Nf1^4F/Arg681*^; DhhCre mice possess a different tumor microenvironment resulting from the nonsense mutation present in all somatic cells. Of note, the neurofibromas arising in Nf1^4F/Arg681*^; DhhCre mice occur in a divergent location when compared with their human counterparts, as these animals develop tumors along the spinal column, whereas symptomatic spinal neurofibromas were absent in humans. This is likely due to the *DhhCre* driver line, which specifically promotes tumorigenesis along the spinal column per previous models (Wu et al., 2008).

Fourthly, there is a great need to develop non-surgical treatments for NF1 patients. To date, most therapeutic interventions that have been tested in preclinical models or clinical trials have been designed to regulate the downstream targets in the Ras signaling pathway or intercellular communication (Yang et al., 2008; Robertson et al., 2012; Jessen et al., 2013). Few studies have focused on restoring function of the defective *NF1* alleles. The development of mutation-guided therapeutics provides new opportunities for treatment of NF1 caused by specific mutations. The robust phenotype of the nmNF1 mouse model and its parallels to human phenotype indicate that Nf1^4F/Arg681*^; DhhCre mice will provide a valuable system for evaluating new therapies targeting the causative genetic mutation, such as nonsense suppression therapies. Nonsense mutations comprise ∼20% of mutations in individuals with NF1 (Messiaen and Wimmer, 2008). For other diseases, therapies (gentamicin, PTC124 and other compounds) have been developed that can suppress nonsense mutations to restore partial protein activity, with some now being used clinically (Du et al., 2002; Keeling et al., 2013; Sangkuhl et al., 2004; Yang et al., 2007; Guerin et al., 2008; Zilberberg et al., 2010). Assessment of nonsense suppression therapy *in vivo* is absolutely required prior to its assessment in individuals with nmNF1. Currently, premature stop mutation mouse models for other genes are being used to evaluate nonsense suppression therapy, including the *Idua-W392X* mouse model of human Hurler syndrome (mucopolysaccharidosis type I-H) and a cystic fibrosis transmembrane conductance regulator model (Zhang et al., 2014; Gunn et al., 2014; Wang et al., 2012). A fundamental question to address for any preclinical nonsense mutation model, including our newly established nmNF1 mouse, is whether sufficient protein activity can be rescued using nonsense suppression therapy approaches.

In summary, we have engineered and characterized the first NF1 patient-based germ line *NF1* mutations modeled in mice, establishing that the Nf1^Arg681*^ allele profoundly reduces neurofibromin expression and leads to plexiform neurofibroma formation. This study provides clear evidence that different mutations in *NF1* can have unique consequences, and the germline mutation is an important factor underlying NF1 clinical heterogeneity. Further preclinical and translational studies of nonsense suppression therapy are underway utilizing the Nf1^Arg681*^ allele. Future investigations of additional NF1 models harboring other human patient mutations will be used to better understand the NF1 phenotype, and will serve as translational models for therapeutic studies.

## MATERIALS AND METHODS

### Study approval

The patient data was collected after approval of a human subjects protocol (F080926009, Further Research on Samples Submitted for Molecular Genetic Testing) by the Institutional Review Board of the University of Alabama at Birmingham. All animal experiments were conducted after approval of an animal protocol (APN 130109835, Translational NF1 Mouse Models) by the Institutional Animal Care and Use Committee of the University of Alabama at Birmingham.

### Generation of Nf1^Arg681*^ and *Nf1^Gly848Arg^* mouse lines

We synthesized two targeting constructs that each contained a point mutation recapitulating human variant c.2041C>T; p.Arg681* (Nf1^Arg681*^) or c.2542G>C; p.Gly848Arg (*Nf1^Gly848Arg^*), a FRT-flanked neomycin selection cassette, and extended 5′ flanking regions homologous to intron 17 or 20, respectively (Genscript, Inc., Piscataway, NJ) (Fig. S1). The truncated versions of the two constructs were introduced into C57BL/6N-tac ES cells (PRX-B6N Primogenix, Laurie, MO) with correctly targeted clones identified by unique PCR fragments. Targeted clones were injected into blastocysts to generate chimeric founder animals that subsequently passed on the targeted alleles to offspring. The excision of the FRT-flanked neomycin cassette was carried out by breeding to a ubiquitously expressing FLP transgene mouse line [B6.129S4-Gt(Rosa)26Sortm1(FLP1), the Jackson Laboratory, #009086]. The new *Nf1* alleles were designated as Nf1^Arg681*^ and *Nf1^Gly848Arg^*. Mice harboring the ‘knockout first NF1 allele (*Nf1^tm1a(KOMP)Wtsi^*) were obtained from the Wellcome Trust Sanger Institute and bred to FLP mice [B6.129S4-Gt(Rosa)26Sortm1(FLP1), the Jackson Laboratory, #009086] to remove the FRT-flanked lacZ-Neo cassettes to produce animals with a conditional knockout allele of the NF1 gene with exon 4 flanked by loxP sites (i.e. *Nf1^tm1c^*, hereafter referred to as *Nf1^4F^*).

### Mouse breeding

All animals were maintained on an inbred C57BL/6 background using a temperature- and humidity-controlled vivarium on a 12 h dark-light cycle with free access to food and water. All homozygous *Nf1^Gly848Arg/Gly848Arg^* and *Nf1^4F/4F^* mice were viable, fertile and did not display any detectable embryonic and postnatal phenotypes. Equal numbers of male and female animals were analyzed throughout the study. Nf1^Arg681*/Arg681*^ and *Nf1^Δ4/Δ4^* embryos failed to develop beyond E9.5. A *DhhCre* transgenic mouse line [FVB(Cg)-Tg(Dhh-cre)1Mejr/J] was kindly provided by Dr Dies Meijer (Erasmus University Medical Center, Netherlands) to breed with the *Nf1^4F/4F^* and Nf1^+/Arg681*^ strains. Cre-negative Nf1^4F/Arg681*^ or *Nf1^4F/4F^* littermates were used for controls. For analysis of embryos, we established timed matings, E0.5 was designated as noon of the day a vaginal plug was detected.

### MEF generation & culture

To generate MEFs, embryos were isolated in cold PBS and the heads and visceral organs were removed. Each embryo was minced into small pieces in trypsin solution and placed at 37°C for 5-10 min. The trypsin solution was then neutralized with two volumes of MEF medium (10% heat-inactivated fetal bovine serum in DMEM). After centrifugation, the supernatant was discarded and the pellet resuspended in fresh MEF medium. The cell suspensions were seeded onto gelatin-coated culture vessels, and incubated at 37°C and 5% CO_2_ in a culture incubator until the cells were confluent and ready for passage. These cells were designated as passage zero (P0), only those P4 or less were used in the study.

### Western blot analysis

Frozen MEFs in culturing vessels or liquid nitrogen-frozen samples of adult brains were lysed in M-PER or T-PER protein extraction reagents containing protease and phosphatase inhibitors (all from Thermo Scientific, Waltham, MA). Protein samples (30 μg) were separated by reducing sodium dodecyl sulfate-polyacrylamide gel electrophoresis and transferred to PVDF membrane. The membranes were then immunoblotted with indicated primary antibodies, followed by incubation with a horseradish peroxidase-coupled anti-rabbit IgG antibody (1:20,000; Jackson ImmunoResearch, West Grove, PA; 711-035-152). Protein bands were visualized with Immobilon Western Chemiluminescent HRP Substrate (EMD Millipore, Billerica, MA). Primary antibodies used for immunoblotting were phospho ERK1/2 and ERK1/2 (1:5000; Cell Signaling Technology, Danvers, MA; 9101 and 9102), β-tubulin (1:5000; Santa Cruz Biotechnology, Santa Cruz, CA; H-235) and neurofibromin (1:500; Santa Cruz Biotechnology; sc-67). Western signal band intensity was quantified using ImageJ Software (National Institutes of Health, Bethesda, MD).

### Nonsense-mediated mRNA decay assay

Total RNA was extracted from brain tissues of adult Nf1^+/Arg681*^ mice using TRIzol reagent (Thermo Scientific). Polyadenylated RNA was reversed transcribed into cDNA in a 20 µl reaction containing 1 μg of total RNA using SuperScript^®^ III First-Strand Synthesis kit (Thermo Scientific). *Nf1* transcripts were amplified around the region of interest using 5 PRIME MasterMix (5 PRIME, Gaithersburg, MD) with 2 µl of the transcribed cDNA as the PCR template. A fragment of 417 bp was amplified with primers forward: 5′-GTTCCTGCGTGCCTGTACTC and reverse: 5′-TGTTCCCACTTTGCATGTGT. Three standard PCR steps were carried out for 30 cycles with 94°C denaturing, 55°C annealing and 72°C extension. The resulting PCR amplicons were cloned into pCR4-TOP vector from the TOP TA cloning kit (Thermo Scientific). Twenty-two individual clones were sequenced using the T7 primer.

### Electron microscopy

Mice of indicated genotypes were intracardially perfused with Karnovsky's fixation solution (3% paraformaldehyde and 3% glutaraldehyde in 0.1 mol/l phosphate buffer, pH 7.4 to 7.6). Sciatic nerves and spinal nerve roots were dissected and post-fixed overnight. Tissues were then transferred to 0.175 mol/l cacodylate buffer, osmicated, dehydrated and embedded. Semi-thin sections were stained in uranyl acetate and lead citrate, and viewed on a FEI Tecnai T12 microscope.

### Histological and immunological analyses

Mice were intracardially perfused with 4% paraformaldehyde (PFA, w/v) in PBS. Neurofibromas, sciatic nerves, spinal nerve roots and other tissues were dissected and post-fixed overnight, followed by cryoprotection and frozen sectioning. Serial sections of 12 μm thickness were prepared using a cryostat (CM3050 S; Leica). Hematoxylin and Eosin (H&E) staining was performed according to standard protocols.

### Statistical analysis

Statistical analyses were performed using GraphPad Prism 5 software (GraphPad Software, Inc., La Jolla, CA). Statistical comparisons were analyzed with one-way ANOVA followed by a Bonferroni post-test. A value of *P*<0.05 was considered statistically significant. Data are presented as mean±s.e.m.
